# Gonococcal resistance to zoliflodacin could emerge via transformation from commensal *Neisseria* species. An in-vitro transformation study

**DOI:** 10.1038/s41598-023-49943-z

**Published:** 2024-01-12

**Authors:** Saïd Abdellati, Jolein Gyonne Elise Laumen, Tessa de Block, Irith De Baetselier, Dorien Van Den Bossche, Christophe Van Dijck, Sheeba Santhini Manoharan-Basil, Chris Kenyon

**Affiliations:** 1 STI Unit, Department of Clinical Sciences, Institute of Tropical Medicine, Antwerp, Belgium; 2 Laboratory of Medical Microbiology, University of Antwerp, Wilrijk, Belgium; 3 Clinical Reference Laboratory, Department of Clinical Sciences, Institute of Tropical Medicine, Antwerp, Belgium; 4 Division of Infectious Diseases and HIV Medicine, University of Cape Town, Cape Town, South Africa

**Keywords:** Microbiology techniques, Molecular medicine, Infectious-disease diagnostics

## Abstract

One of the most promising new treatments for gonorrhoea currently in phase 3 clinical trials is zoliflodacin. Studies have found very little resistance to zoliflodacin in currently circulating *N. gonorrhoeae* strains, and in-vitro experiments demonstrated that it is difficult to induce resistance. However, zoliflodacin resistance may emerge in commensal *Neisseria* spp., which could then be transferred to *N. gonorrhoeae* via transformation. In this study, we investigated this commensal-resistance-pathway hypothesis for zoliflodacin. To induce zoliflodacin resistance, ten wild-type susceptible isolates belonging to 5 *Neisseria* species were serially passaged for up to 48 h on gonococcal agar plates containing increasing zoliflodacin concentrations. Within 7 to 10 days, all strains except *N. lactamica*, exhibited MICs of ≥ 4 µg/mL, resulting in MIC increase ranging from 8- to 64-fold. The last passaged strains and their baseline were sequenced. We detected mutations previously reported to cause zoliflodacin resistance in GyrB (D429N and S467N), novel mutations in the quinolone resistance determining region (QRDR) (M464R and T472P) and mutations outside the QRDR at amino acid positions 28 and 29 associated with low level resistance (MIC 2 µg/mL). Genomic DNA from the laboratory evolved zoliflodacin-resistant strains was transformed into the respective baseline wild-type strain, resulting in MICs of ≥ 8 µg/mL in most cases. WGS of transformants with decreased zoliflodacin susceptibility revealed presence of the same zoliflodacin resistance determinants as observed in the donor strains. Two inter-species transformation experiments were conducted to investigate whether zoliflodacin resistance determinants of commensal *Neisseria* spp. could be acquired by *N. gonorrhoeae*. *N. gonorrhoeae* strain WHO P was exposed to (i) pooled genomic DNA from the two resistant *N. mucosa* strains and (ii) a *gyrB* amplicon of the resistant *N. subflava* strain 45/1_8. Transformants of both experiments exhibited an MIC of 2 µg/mL and whole genome analysis revealed uptake of the mutations detected in the donor strains. This is the first in-vitro study to report that zoliflodacin resistance can be induced in commensal *Neisseria* spp. and subsequently transformed into *N. gonorrhoeae.*

## Introduction

The continued emergence and spread of combined gonococcal resistance to ceftriaxone and azithromycin has reinvigorated the search for alternative therapies^1^. One of the most promising new treatments currently in phase 3 clinical trials is zoliflodacin (NCT03959527)^1^. Zoliflodacin (ETX0914) belongs to a new class of antibiotics that inhibit bacterial DNA replication via interactions with DNA gyrase subunit B^2^. Studies have found very little resistance to zoliflodacin in currently circulating strains of *Neisseria gonorrhoeae*^3^. In addition, a number of in-vitro experiments have found that it is difficult to induce zoliflodacin resistance in *N. gonorrhoeae* using currently recommended treatment regimens^4–6^. Based on these findings, several authors have concluded that there is a low probability of zoliflodacin resistance emerging in *N. gonorrhoeae* following its introduction as a treatment for gonorrhoea^3,4,6^.

We and others have hypothesized that zoliflodacin resistance may emerge in commensal *Neisseria* spp. which could then be transferred to *N. gonorrhoeae* via transformation^7^. This hypothesis was based on the fact that transformation of resistance from commensal *Neisseria* spp. into *N. gonorrhoeae* has been instrumental in the emergence of cephalosporin, sulfonamide and macrolide resistance^8–11^. Transformation of *gyrA* has also played an important role in the genesis of fluoroquinolone resistance in *N. meningitidis*. A study in Shanghai, found that 99.3% of commensal *Neisseria* and 67.7% of *N. meningitidis* isolates were resistant to fluoroquinolones and that horizontal gene transfer (HGT) from commensals was responsible for fluoroquinolone resistance in over half the *N. meningitidis* isolates^12^. An in silico analysis of 20,047 *Neisseria* isolates from around the world found evidence that a number of strains of *N. gonorrhoeae* have previously taken up sections of *gyrB* from commensal *Neisseria*, including the quinolone resistance-determining region (QRDR; 1255–1488 bp) of *gyrB*, which is also the zoliflodacin resistance-conferring region^13^.

In vitro studies have demonstrated that gonococcal zoliflodacin resistance typically emerges via three substitutions in GyrB—Asp429Asn, Lys450Thr or Ser467Asn^2,6^. These findings provided the justification for the current paper where we investigated the commensal-resistance-pathway hypothesis as follows: Firstly, we sought to assess the zoliflodacin MICs of circulating commensal *Neisseria* species. Secondly, we assessed if we could induce zoliflodacin resistance in *N. cinerea, N. lactamica, N. macacae*, *N. mucosa* and *N. subflava*^14^. Thirdly, we evaluated which *gyrB* mutations were associated with evolved decreased susceptibility to zoliflodacin in these species. Finally, we attempted to transform zoliflodacin resistance-conferring DNA within and between *Neisseria* species including from *N. subflava*/*N. mucosa* into *N. gonorrhoeae*.

## Methods

### Sampling, ethical approval and bacterial isolates used in the study

Details of the isolates used for this experiment are provided in Tables 1, 2 and Suppl Table 1. The commensal *Neisseria* isolates were cultured from oropharyngeal swabs from asymptomatic men and women participating in three recently performed clinical studies at our centre in Belgium—the Resistogenicity, ComCom, PReGo and ResistAZM (NCT05027516) studies^14–16^. The samples were obtained between 2019 and 2022. Written informed consent was obtained from all participating patients and the studies were approved by the Institute of Tropical Medicine’s Institutional Review Board (1276/18 and 1351/20) and from the Ethics Committee of the University of Antwerp (19/06/058 and AB/ac/003). Our selection of isolates from these studies was biased towards *N. mucosa* and *N. macacae,* as in a previous phylogenetic study of 20, 047 *Neisseria* isolates, we found that these were the only two commensal *Neisseria* species which were donors of *gyrB* DNA into *N. gonorrhoeae*^13^. We also included a number of *Neisseria* isolates from reference collections (Suppl Table 1).

**Table 1:** Characteristics of strains used for transformation and induction of resistance experiments.

Isolate	Species	Zoliflodacin MIC (mg/L)*	Azithromycin MIC (mg/L)	Ceftriaxone MIC (mg/L)	Ciprofloxacin MIC (mg/L)	Source of isolate
9/1	*N. subflava*	0.5	3	0.023	ND	Resistogenicity study^15^
45/1	*N. subflava*	2	6	0.047	ND	Resistogenicity study
773/3	*N. mucosa*	0.125	2	0.032	0.006	PREGO/ComCom study^16^
801/1	*N. mucosa*	1	ND	ND	ND	PREGO/ComCom study^16^
DSM4631	*N. mucosa*	0.5	ND	ND	ND	Reference strain
793/3	*N. macacae*	0.5	ND	ND	ND	PREGO/ComCom study^16^
782/1	*N. cinerea*	0.125	2	< 0.016	0.012	PREGO/ComCom study^16^
761/1	*N. lactamica*	0.125	2	< 0.016	0.19	PREGO/ComCom study^16^
ATCC49226	*N. gonorrhoeae*	0.125	0.25	< 0.016	0.003	Reference strain
WHO F	*N. gonorrhoeae*	0.032	0.125	< 0.016	0.004	WHO reference strain^34^
WHO P	*N. gonorrhoeae*	0.125	4	0.004	0.004	WHO reference strain^34^
WHO X	*N. gonorrhoeae*	0.125	0.25	1.5	> 32	WHO reference strain^34^
WHO Z	*N. gonorrhoeae*	0.125	0.75	0.5	> 32	WHO reference strain^34^
38/1	*N. gonorrhoeae*	0.125	0.25	0.016	ND	Resistogenicity study^15^

*ND* not determined.

*MIC’s determined in the present study.

**Table 2:** Intraspecies transformation of DNA conferring reduced susceptibility to zoliflodacin.

Initial strain			Sample_ID	SRA run no	Resistant strain	GyrB substitution (AA)	Nucleotide [nr of reads (%)]	Transformant	
Species	Sample_ID	Initial MIC (µg/mL)			Final MIC (µg/mL)			gyrB mutation transformant [nr of reads (%)]	MIC (µg/mL)
*Neisseria subflava*	9/1	0.5	9/1_8^#^	SRR23198175	8	G28C	G82T (581/584 (99%))	NS	2
*Neisseria subflava*	45/1	2	45/1_8^#^	**SRR23198182**	≥ 16	D429N	G1285A (977/983 (99%))	D429N (696/697 (100%))	8
*Neisseria mucosa*	773/3	0.25	773/3_8^#^	**SRR23198181**	≥ 16	M464R	T1391G (898/901 (100%))	M464R (523/524 (100%))	≥ 16
*Neisseria mucosa*	801/1	2	801/1_8^#^	**SRR23198179**	≥ 16	K450I	Heteroresistance	K450I–A1349T (703/706 (100%))	≥ 16
*Neisseria mucosa*	DSM4631	0.5	DSM4631_8^#^	**SRR23198178**	≥ 16	T472P	A1414C (782/1195 (65%))	No mutations in gyrB	≥ 16
*Neisseria macacae*	793/1	0.5	793/1		4	NS	NS	NS	NS
*Neisseria cinerea*	782/1	0.25	782/1_8^#^	**SRR23198180**	8	S467G	A1399G (956/958 (100%))	S467G (645/646 (100%))	8
*Neisseria gonorrhoeae*	ATCC49226	0.25	ATCC49226_8^#^	SRR23198175	8	S467N	G1400A (1107/1109 (100%)	NA**	NA
*Neisseria gonorrhoeae*	WHO P	0.125	WHO P_8^#^	**SRR23198175**	8	M29I	G87A (1481/1484 100%)	S467N (945/947 (100%)**	2

*NS* not sequenced, *NA* not applicable.

^#^Isolates that were whole genome sequenced.

**Pool: ATCC49226_8 and WHO-P_8 as donor and WHO-P as recipient.

Significant values are in bold.

All methods were performed in accordance with the relevant guidelines and regulations. Briefly, suspensions from tenfold dilutions of the oropharyngeal swabs in PBS were inoculated on *Neisseria* commensal selective agar plates (LBVT.SNR). Species identity was confirmed via MALDI-TOF and whole genome sequencing (WGS) was carried out as detailed elsewhere^17^.

### In vitro assays

#### Determination of the MIC

All the frozen isolates stored in skimmed milk at − 80 °C were revived on gonococcal base (GCB) agar (Gonococcal Medium Base, BD Difco™) supplemented with 1% IsoVitaleX (BD BBL™) and subcultured twice before starting the experiments.

Minimal inhibitory concentrations (MIC ≤ 0.015 to 16 µg/mL) for zoliflodacin (obtained from MedChemExpress) were determined on GCB agar in accordance with the CLSI methodology^18^. The WHO gonococcal reference strains F, P, X, Z and the ATCC strain 49,226 were included as the control isolates. The bacterial inoculum size was 10^4^ colony-forming units. The inoculated plates were incubated at 36 °C in 5% CO_2_ with high humidity. MICs were read after 24 h of incubation.

#### Serial passage experiments

Briefly, the strains were inoculated on a GCB agar plate containing 0.015 mg/L zoliflodacin and incubated at 36 °C in an atmosphere of 5% CO_2_. After visible growth was attained, colonies from the GCB agar plate with 0.015 mg/L zoliflodacin were inoculated onto a GCB agar plate with a twofold higher zoliflodacin concentration compared to the previous plate (0.03 mg/L). This process was repeated for each strain until no visible growth was seen on cultured plates or growth was obtained on the plate with the final concentration of 16 µg/mL. The cultures from each time point were stored in skimmed milk (Skim Milk, BD Difco™) supplemented with 20% of glycerol and stored at − 80 °C.

### Genomic DNA extraction and fragmentation

Suspensions of bacteria from overnight cultures on GC agar plates were prepared in 2 mL of phosphate buffered saline (PBS; pH 7.6). Genomic DNA extraction was carried out using the QIAamp DNA Mini kit (Qiagen, Hilden, Germany) following the manufacturer’s protocol for isolation of genomic DNA from bacterial cultures. DNA was eluted in a total volume of 300 µL Aqua SteropFlexo (Sterop group, Belgium) and stored at 4 °C for further use. The DNA concentration and purity was determined using the Nanodrop® ND-1000 spectrophotometer (Thermo Scientific, Waltham, MA, USA). A concentration of 100 ng/µL was used in subsequent experiments.

Samples of purified genomic DNA (gDNA) were sheared into short fragments using ultrasonication shearing to be used in transformation experiments^19^. After sonication, all samples were stored at 4 °C. The size of the gDNA fragments were assessed with the Agilent Tape station (Agilent Technologies, Waldbronn, Germany) and visualized on 1% agarose gel^20^.

### PCR amplification of *gyrB*

PCR amplification of a 253 bp *gyrB* that included the resistance mutation at position D249 were amplified using the primer pairs gyrB-F1-1118-1353_Zoli (5ʹ-TCATCACCARCAAAATCGTC-3ʹ) and gyrB-R1-1118-1353_Zoli (5ʹ-ACCTTTGAGCGGCAAAATC-3ʹ). The primers were synthesized by Eurogentec (Seraing, Belgium). The PCR mixture consisted of 1× PE Buffer (Perkin–Elmer, Cetus, Norwalk, CT, USA), 2 mM MgCl_2_, 0.28 mM deoxyribonucleoside triphosphate (dNTP) (Pharmacia Biotech, St Albans, UK), 0.15 μM of primers, and 2 U of Taq polymerase (Perkin–Elmer, Cetus, Norwalk, CT, USA). 20 ng of DNA extract was added to the reaction mixture.

A one-step thermocycling protocol was carried out in a thermocycler (Perkin–Elmer, Cetus, Norwalk, CT, USA) as follows: Initial denaturation at 94 °C for 10 min, followed by 35 cycles of denaturation at 94 **°**C for 45 s (s), annealing at 54 °C for 45 s, and extension at 72 °C for 1 min (min). The final extension step was carried out at 72 °C for 5 min. The PCR amplicon (15 μL) was visualized via electrophoresis in a 1% agarose gel in 1× Tris–acetate-EDTA buffer (pH 8.5). The gel was stained with Gelred (0.5 mg/L; Sigma, Bornem, Belgium) and was photographed under short-UV light. The size of the amplified products was assessed by comparing with a 100 bp Smartladder marker (Eurogentec).

### Intra- and inter-species transformation

#### Transformation of reduced zoliflodacin susceptibility isolates with fragmented genomic DNA

For intra-species transformation, the genomic DNA from the resistant strains that were generated via serial passage experiments were transformed into the susceptible wild-type strains from which the resistant strains were evolved (Table 2). For intra-species transformation, the WHO P *N. gonorrhoeae* recipient strain was transformed with genomic DNA from pools of two commensal strains each (Table 3). Transformations were conducted as described in Ref.^8^. Briefly, the strains were suspended in GCB broth (15 g/L protease peptone 3, 1 g/L soluble starch, 4 g/L dibasic K2HPO4, 1 g/L monobasic KH_2_PO_4_, 5 g/L NaCl) supplemented with 1% isovitalex, 10 µM MgSO_4_ and 2.5 µg of fragmented gDNA. The suspensions were incubated at 37 °C for 1h and plated on non-selective GCB agar plates overnight. The recovered transformants were then placed on selective GCB plates with 0.125, 1, 2 and 4 µg/mL of zoliflodacin for 18–24 h.

**Table 3:** Interspecies transformation of DNA conferring reduced susceptibility to zoliflodacin.

Recipient			Donor			Transformant	
Species	Sample_ID	MIC	Species	Sample_ID	MIC	GyrB substitution	MIC
A. Transformation with genomic DNA							
Neisseria gonorrhoeae	WHO P	0.125	Neisseria mucosa	801/1_8	8	K450N	2
				DSM4631_8	8		
			Neisseria subflava	45/1_16	≥ 16	No growth	NA
				9/1_8	8	No growth	NA
			Neisseria mucosa/macacae	773/2_ ≥ 16	≥ 16	No growth	NA
				793/1_4	4	No growth	NA
			Neisseria cinerea	782/1_8	8	No growth	NA
Neisseria gonorrhoeae	Res18	0.125	Neisseria mucosa	801/1_8	8	No growth	NA
				DSM4631_8	8	No growth	NA
			Neisseria subflava	45/1_16	≥ 16	No growth	NA
				9/1_8	8	No growth	NA
			Neisseria mucosa/macacae	773/2_ ≥ 16	≥ 16	No growth	NA
				793/1_4	4	No growth	NA
			Neisseria cinerea	782/1_8	8	No growth	NA
B. Transformation with amplicon DNA							
Neisseria gonorrhoeae	WHO P	0.125	Neisseria subflava	45/1_8	≥ 16	D429N	2

#### Transformation of PCR amplicon with zoliflodacin mutation

For intra- and inter-species transformation, the recipient strains were transformed with ~ 2.5 µg of PCR-purified products and the transformation was carried out as described above. The experiment was carried out for limited isolates (Table 3, Suppl Table 2).

### Whole genome sequencing and analysis

The whole genome data available from the Resistogenicity, ComCom and PReGo studies were included in this study (Suppl Table 1). In addition, eight isolates, including isolates with zoliflodacin resistance determining mutations with confirmed phenotypic resistance from the serial passage (highest obtained MIC) and transformation experiments, as well as their wild-type baselines, were subjected to WGS (Table 2). WGS was outsourced to Eurofins (Konstanz, Germany). The Dneasy® Blood & Tissue Kit (Qiagen, Hilden, Germany) was used to extract genomic DNA, which was then suspended in nuclease-free water (Sigma-Aldrich, Seelze, Germany). Library preparation was carried out using Nextera XT DNA library prep kit followed by Illumina sequencing using paired-end 150-bp read sequencing chemistry (Illumina, San Diego, CA, USA). Raw reads that were generated were quality-controlled using FastQC (v0.11.9) and trimmed using Trimmomatic (v0.39)^21,22^. Shovill (v1.0.4, https://github.com/tseemann/shovill) was used to de novo assemble the wild-type baseline isolates, which were subsequently annotated with Prokka (v1.14.6)^23^. The consensus *gyrB* gene, encoding the DNA gyrase subunit B, was extracted, and reads of the isolates exhibiting zoliflodacin decreased susceptibility were mapped to their baseline using the Burrows-Wheeler Alignment Tool (v0.7.17-r1188) and Samtools (v1.9)^24^. Reference mappings were visualized using the Integrative Genomics Viewer (IGV, v2.5.3) to discover single-nucleotide polymorphisms (SNPs) compared to the baseline and to estimate the proportion of reads mutated^25^. Multiple sequence alignments of the *gyrB* were generated using MEGA software^26^. The raw reads are deposited at https://www.ncbi.nlm.nih.gov/sra/PRJNA926517.

**Figure 1:**
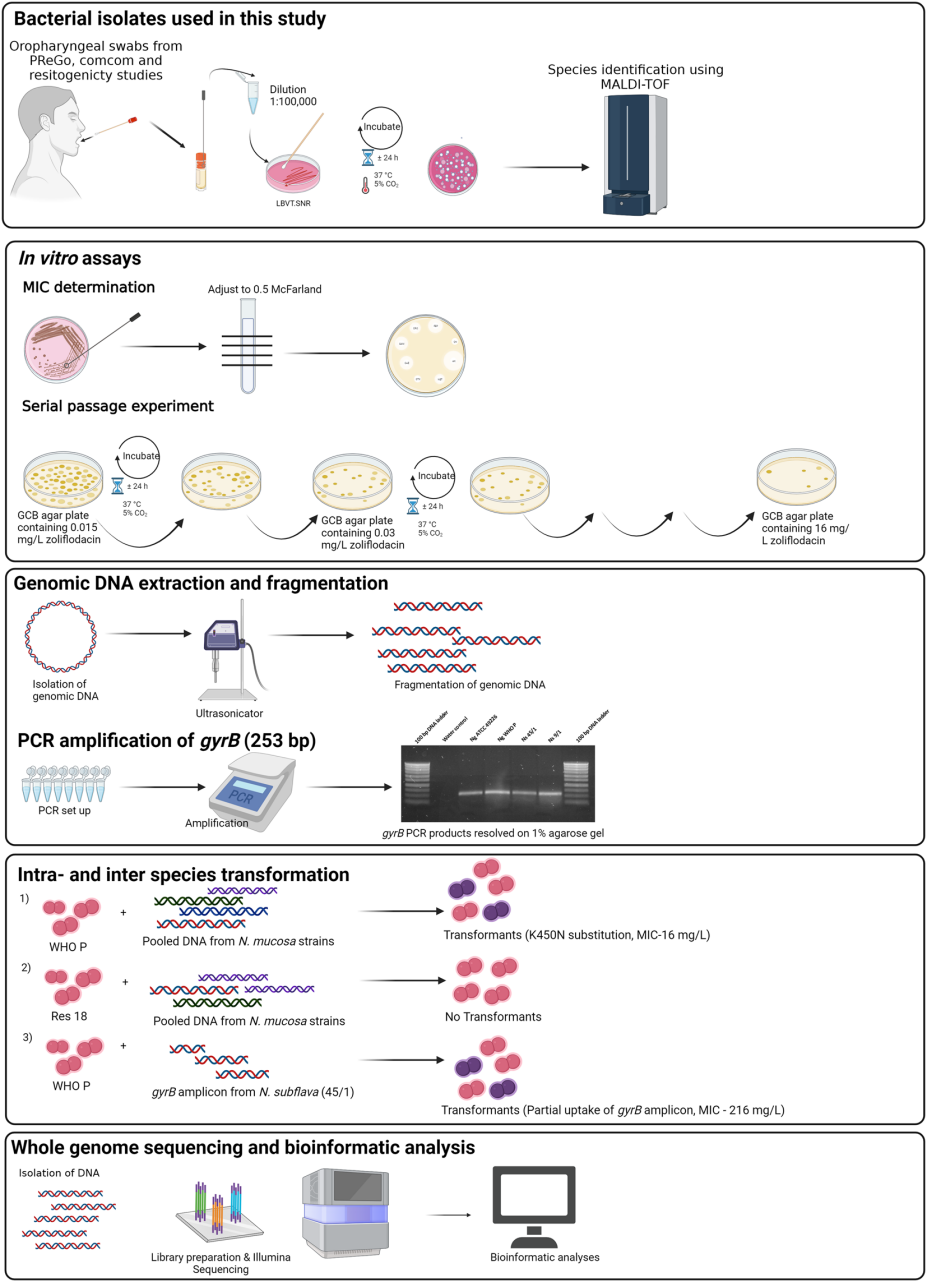
Overview of the study. The figure was created using BioRender.

The overview of the study is illustrated in Fig. 1.

### Statistics

Differences in MICs between groups were assessed using the Mann–Whitney test. Statistical analyses were performed using GraphPad Prism v9.

## Results

### Baseline MICs of *Neisseria* species isolates

**Figure 2:**
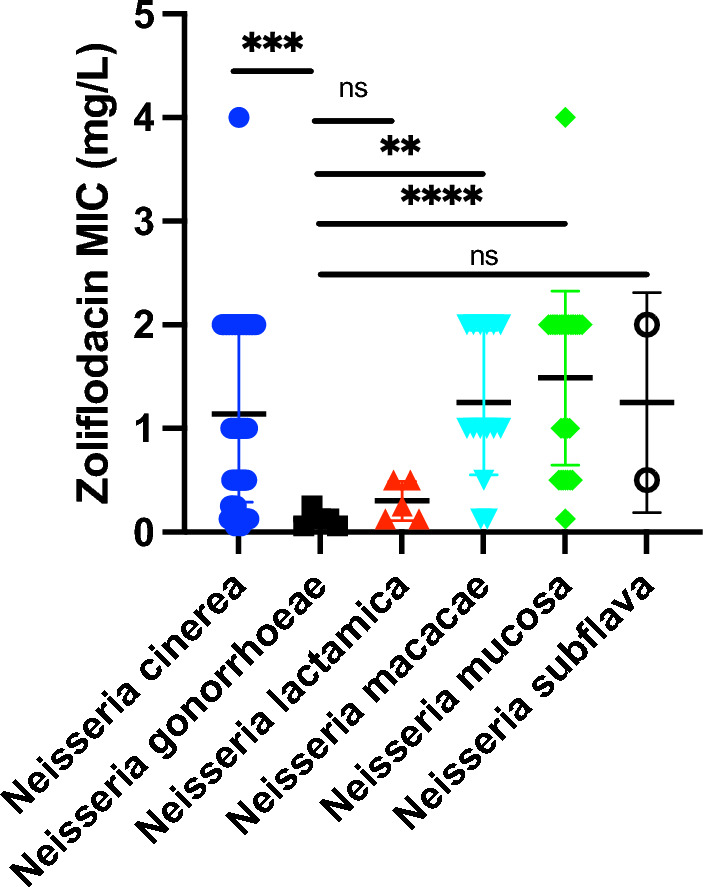
Zoliflidacin MICs of 62 strains from 5 *Neisseria* species (**P < 0.001; ***P < 0.0001; *ns* non-significant).

In the absence of a breakpoint for zoliflodacin resistance, we classified a MIC of ≥ 4 µg/mL as resistantThe zoliflodacin MICs of *N. cinerea* (median 1 µg/mL, IQR 0.5–2 µg/Ml, n = 4; P < 0.0005), *N. macacae* (median 1 µg/mL, IQR 1–2 µg/mL, n = 15; P < 0.005), and *N. mucosa* (median 2 µg/mL, IQR 0.5–2 µg/mL, n = 30; P < 0.0005), were higher than those of *N. gonorrhoeae* (median 0.125 µg/mL, IQR 0.06–0.19 µg/mL, n = 5; Fig. 2; Suppl Table 1). The MICs of *N. lactamica* (median 0.25 µg/mL, IQR 0.125–0.5 µg/mL, n = 5) were similar to those of *N. gonorrhoeae*. The sample size for *N. subflava* was too small (n = 2) to warrant statistical testing.

### Induction of zoliflodacin resistance

**Figure 3:**
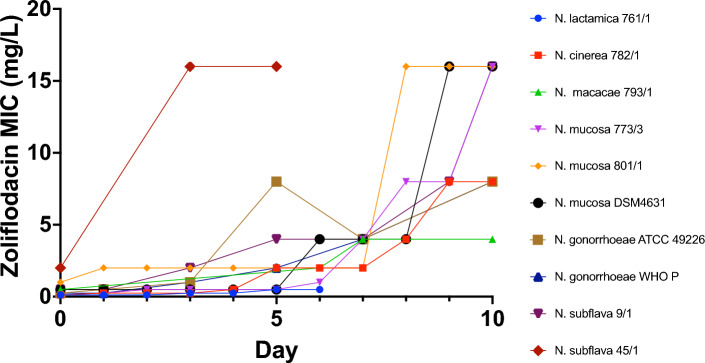
Increase of zoliflodacin MICs during passage experiments using increasing concentrations of zoliflodacin for 10 strains from 5 *Neisseria* species. Zoliflodacin MICs were truncated at 16 µg/mL.

To induce zoliflodacin resistance, ten susceptible *Neisseria* isolates [*N. lactamica* (n = 1), *N. cinerea* (n = 1), *N. macacae* (n = 1), *N. subflava* (n = 2), *N. mucosa* (n = 3)*,* and *N. gonorrhoeae* (n = 2)] were serially passaged on agar plates containing increasing zoliflodacin concentrations (Fig. 3). Within 7 to 10 days, all strains, except the *N. lactamica* strain, attained MICs of 4 µg/mL or higher, resulting in MIC increases ranging from 8- to 64-fold (Supplemental Table 1). There was only a two-fold increase in MIC for the *N. lactamica* strain (Fig. 3). The increase in MIC was most rapid in *N. subflava* 45/1, with the MIC increasing from 2 to ≥ 16 µg/mL in 3 days. The minimum time required for the emergence of zoliflodacin resistance was thus 3 days. The last passaged strains from the passage experiment and their baselines were subjected to WGS. In all strains, in vitro*-*induced zoliflodacin resistance was associated with mutations in the *gyrB* gene (Table 2). In *N. subflava* and *N. gonorrhoeae,* we detected mutations previously reported to cause zoliflodacin resistance, i.e. D429N and S467N. At position 467, we also identified a serine to glycine amino acid substitution in a *N. cinerea* strain, rather than the 467-asparagine resistance mutant described in the literature. Although we did not find the well-known K450N/T mutation, the consensus sequence reported an Isoleucine at position 450 in a *N. cinerea* strain. However, reference mapping revealed heteroresistance at this position, with 75% of the reads corresponding to K450I and 25% leading to K450N substitutions. Novel mutations, M464R and T472P, were discovered in the quinolone resistance-determining region (QRDR) of *N. mucosa*. Interestingly, we also observed novel mutations at amino acid positions 28 and 29 outside the QRDR in *N. subflava* and *N. gonorrhoeae,* respectively, that were the only genetic variants detected in *gyrB* in the resistant strains compared to their wild-type baseline. We found no evidence of multiple or multi-step mutations in *gyrB*.

### Intra-species transformation of zoliflodacin resistance determinants

The genomic DNA of the resistant strains was transformed into their own susceptible baseline strain. This procedure resulted in zoliflodacin MICs of 8 µg/mL or higher (Table 2). WGS of transformants with decreased zoliflodacin susceptibility revealed the presence of the same zoliflodacin resistance determinants as observed in the donor (Table 2). The WHO-P *N. gonorrhoeae* reference strain was exposed to a pool of genomic DNA of zoliflodacin-resistant *N. gonorrhoeae* WHO-P and ATCC strain 49226. Only the mutation found in the ATCC strain (S467N) was detected in the transformant’s sequence analysis (Table 2). Attempts to transform the PCR product of *gyrB* from zoliflodacin resistant *N. gonorrhoeae* ATCC 49226_8 and WHO-P_8 into *N. gonorrhoeae* WHO-P failed (Table S2).

### Inter-species transformation of zoliflodacin resistance determinants

Three inter-species transformation experiments were conducted to investigate whether zoliflodacin resistance determinants of commensal *Neisseria* could be acquired by *N. gonorrhoeae*. *N. gonorrhoeae* reference strain WHO-P was exposed to pooled genomic DNA from the three resistant *N. mucosa* strains, resulting in a MIC of ≥ 16 µg/mL and uptake of the previously reported K450N mutation present in a proportion of the reads of the heteroresistant donor strain 801/1_8 (Table 3). No evidence of transformation was detected in equivalent experiments with the same donor strains but with *N. gonorrhoeae*, Res 18 as the recipient (Table 3).

**Figure 4:**
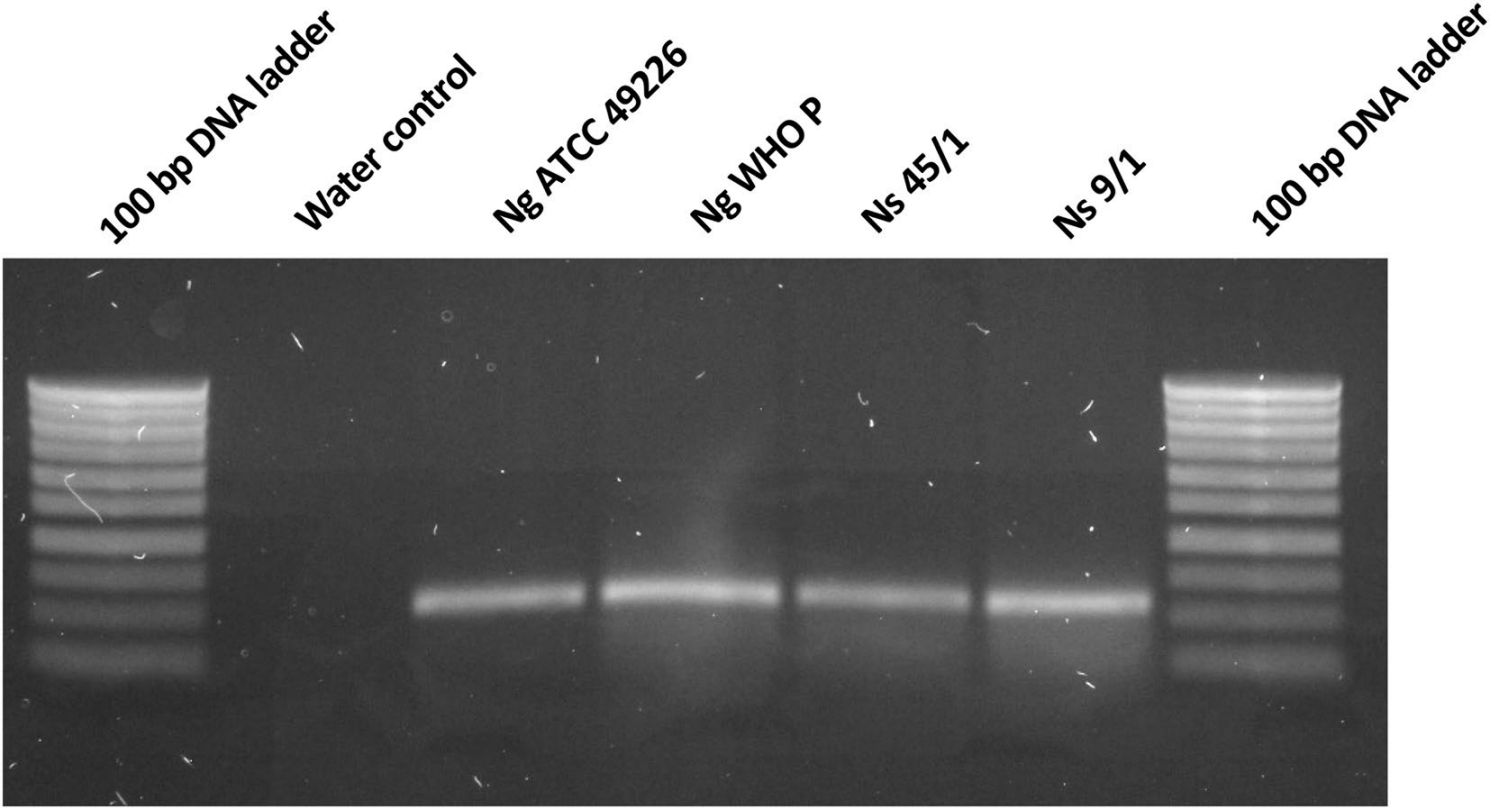
An image showing the PCR amplified *gyrB* (235 bp) in 1% agarose gel electrophoresis. *GyrB* was amplified from DNA of Ng (*Neisseria gonorrhoeae*; n = 2) and Ns (*Neisseria subflava*; n = 2). Milli Q. H_2_O was was used as a negative control.

In the third experiment, the *gyrB* amplicon (253 bp) (Fig. 4) of the zoliflodacin resistant *N. subflava* strain 45/1_8 was used as the donor, and *N. gonorrhoeae* WHO-P as the recipient. The zoliflodacin MIC of *N. gonorrhoeae* WHO-P showed an increase from 0.125 to 216 µg/mL, and sequence alignment revealed that it had taken up a part of the gyrB amplicon of the resistant *N. subflava* strain 45/1_8 (Table 3, Fig. S1).

### *gyrB* mutations in circulating commensal *Neisseria* species

We found 7 mutations in *gyrB* that emerged in response to zoliflodacin selection pressure. We assessed if any of these mutations were present in the zoliflodacin MIC panel of commensal *Neisseria* species. None of these strains had any of these mutations. We did however find a different single amino acid substitution at position 472. T472P mutation that was followed by a 32-fold increase in zoliflodacin MIC, emerged in *N. mucosa* DSM4631. We found two strains of *N. lactamica* (CO000761/1, CO000771/1) and three strains of *N. cinerea* (CO000776/4, CO000782/1) that had T472A substitution. This single amino acid change was not associated with zoliflodacin MICs (Mann–Whitney test, P > 0.05 for both species).

## Discussion

We found that the zoliflodacin MICs of *N. cinerea*, *N. macacae* and *N. mucosae* were higher than those of *N. gonorrhoeae.* Our passage experiments revealed that zoliflodacin resistance could be induced in all species assessed—an 8- to 64-fold increase within 7 to 10 days. Finally, we were able to transform zoliflodacin resistance both within and between *Neisseria* species. Of particular concern, transformation experiments revealed that *N. gonorrhoeae* could obtain zoliflodacin resistance from *N. subflava*—the most prevalent commensal *Neisseria* species.

As noted above, transformation of resistance from commensal *Neisseria* spp. into *N. gonorrhoeae* has been instrumental in the emergence of cephalosporin, sulfonamide and macrolide resistance^8–11^. Transformation from commensal *Neisseria* has also been found to be responsible for fluoroquinolone resistance in over half the *N. meningitidis* isolates in one study from China^12^. Our findings support the possibility that gonococcal resistance to zoliflodacin could emerge via resistance in commensal *Neisseria*. Because commensal *Neisseria* such as *N. subflava* are a key component of a healthy oropharyngeal microbiome they are present in almost all individuals^14,27^. This high prevalence means they are at considerably greater risk for bystander selection—the emergence of AMR in response to antimicrobials taken for other indications^28^. The broader the range of infections that zoliflodacin is used to treat, the higher the risk of this bystander selection. Even if the use of zoliflodacin is restricted to STIs such as *N. gonorrhoeae* and *M. genitalium*, the high prevalence of these infections in high risk groups such as HIV PrEP cohorts (around 10–15% for both infections^29,30^), will translate into high zoliflodacin exposure within these groups. The higher prevalence of commensal than pathogenic *Neisseria* in these groups will mean that the commensals are under a greater selection pressure to acquire resistance to zoliflodacin. This effect may be particularly prominent for zoliflodacin on commensal *Neisseria* as the penetration of zoliflodacin in the oropharynx appears to be particularly poor. Poor oropharyngeal penetration of zoliflodacin is the most plausible explanation for the lower cure rates for gonorrhoea in the pharynx (50%) than urogenital sites (96%)^1^.

In this study we found that the zoliflodacin MICs of *N. mucosa*, *N. macacae* and *N. cinerea* were higher than those in *N. gonorrhoeae*. These higher MICs could not be explained by established *gyrB* resistance associated mutations. It should also be noted that other studies have found that the known zoliflodacin resistance associated mutations are not able to fully explain differences in zoliflodacin MICs. For example, a study of 986 gonococcal isolates collected in China between 2014 and 2018 found a doubling in MIC_50_ and MIC_90_ over the time period, but no known zoliflodacin RAMs were detected^31^. We found that zoliflodacin resistance can emerge fairly rapidly within commensal *Neisseria* species and be transformed into *N. gonorrhoeae*. These results suggest it may be prudent to include surveillance of zoliflodacin susceptibility in commensal *Neisseria* in clinical trials and programmes using this agent. This suggestion would fit into calls to include surveillance of antimicrobial susceptibility of commensal Neisseria within gonococcal surveillance programmes such as Euro-GASP^7,32^.

There are a number of limitations to this study. Our study only used a limited number of strains from a small number of commensal species. We did not include *N. meningitidis*. The main *gyrB* mutations we found to be implicated in zoliflodacin resistance are well-established in previous studies. We did however find two additional *gyrB* mutations which may increase zoliflodacin MICs. However, we were unable to prove this effect experimentally. Additionally, we would like to acknowledge that several aspects were not assessed in our study. We did not determine the mutation frequency and the stability of the induced zoliflodacin resistance, assess the transformation efficacy, explore potential fitness effects associated with induced zoliflodacin resistance, or investigate cross-resistance to other antimicrobials. These are important considerations that should be addressed in future studies to gain a more comprehensive understanding of zoliflodacin resistance. Furthermore, it is crucial to acknowledge that in vitro induced mutations may not fully represent the mutations that develop in vivo. While our study provides insights into the potential mechanisms of zoliflodacin resistance, the translation of these findings to clinical settings requires further investigation.

Transformation efficacy between *Neisseria* spp. is heavily influenced by the similarity of the direct uptake sequences (DUS) of the recipient and donor^33^. Analyses have revealed the existence of eight families of DUS sequence with the *Neisseriaceae* family^33^. *N. gonorrhoeae* is from the same DUS family (AT-DUS) as *N. meningitidis, N. lactamica, N. polysaccharea* and *N. cinerea*^33^. The *N. mucosa* and *N. subflava* used in the transformation experiments are from the closely related to AG-DUS family^33^. Thus the fact that the donor species used in the transformation experiments were from a different DUS family to *N. gonorrhoeae* may be considered as further study limitation.

Notwithstanding these limitations, this is the first study to report that zoliflodacin resistance can be induced in commensal *Neisseria* and subsequently acquired by *N. gonorrhoeae* via transformation. This finding has important implications for how we introduce this novel antimicrobial and how we monitor for the emergence of zoliflodacin resistance.

### Supplementary Information

Supplementary Information.

## Data Availability

All data generated or analysed during this study are included in this published article and its Supplementary Information files. Please see: https://www.ncbi.nlm.nih.gov/sra/PRJNA926517.
