# Supplementary material for: Gonococcal resistance to zoliflodacin could emerge via transformation from commensal Neisseria species. An in-vitro transformation study
Source: Sci Rep. 2024 Jan 12;14:1179. doi: 10.1038/s41598-023-49943-z (PMC10786824; doi:10.1038/s41598-023-49943-z)
Supplement: Supplementary file 1 — Supplementary Information. [file 41598_2023_49943_MOESM1_ESM.docx]

**Supplementary Tables 1 and 2, and Supplementary Figure 1**

STable 1: List of all isolates used in the study

| Study | Species | Isolate ID | International Ref. ID | Zoliflodacin MIC | WGS | T472A | SRA run no |
| --- | --- | --- | --- | --- | --- | --- | --- |
| COMCOM1 | *Neisseria cinerea* | Co000769/3 |  | 0.25 | Yes | 0 | 1 |
| COMCOM1 | *Neisseria cinerea* | Co000776/4 |  | 1 | Yes | 1 | 1 |
| COMCOM1 | *Neisseria cinerea* | Co000782/1 |  | 0.125 | Yes | 1 | 1 |
| COMCOM1 | *Neisseria lactamica* | Co000761/1 |  | 0.125 | Yes | 1 | 1 |
| COMCOM1 | *Neisseria lactamica* | Co000771/1 |  | 0.125 | Yes | 1 | 1 |
| COMCOM1 | *Neisseria mucosa* | Co000770/5 |  | 1 | Yes | 0 | 1 |
| COMCOM1 | *Neisseria mucosa* | Co000771/4 |  | 2 | Yes | 0 | 1 |
| COMCOM1 | *Neisseria mucosa* | Co000772/3 |  | 2 | Yes | 0 | 1 |
| COMCOM1 | *Neisseria mucosa* | Co000773/3 |  | 0.125 | Yes | 0 | 1 |
| COMCOM1 | *Neisseria mucosa* | Co000779/1 |  | 1 | Yes | 0 | 1 |
| COMCOM1 | *Neisseria mucosa* | Co000783/3 |  | 2 | Yes | 0 | 1 |
| COMCOM1 | *Neisseria mucosa* | Co000783/6 |  | 2 | Yes | 0 | 1 |
| COMCOM1 | *Neisseria mucosa* | Co000787/3 |  | 2 | Yes | 0 | 1 |
| COMCOM1 | *Neisseria mucosa* | Co000788/1 |  | 2 | Yes | 0 | 1 |
| COMCOM2 | *Neisseria macacae* | Co00791/3 |  | 1 | Yes | NA | 0 |
| COMCOM2 | *Neisseria macacae* | Co00792/3 |  | 1 | Yes | NA | 0 |
| COMCOM2 | *Neisseria macacae* | Co00793/1 |  | 0.5 | Yes | NA | 0 |
| COMCOM2 | *Neisseria macacae* | Co00794/2 |  | 0.125 | Yes | NA | 0 |
| COMCOM2 | *Neisseria macacae* | Co00794/3 |  | 0.125 | Yes | NA | 0 |
| COMCOM2 | *Neisseria macacae* | Co00803/2 |  | 2 | Yes | NA | 0 |
| COMCOM2 | *Neisseria macacae* | Co00804/2 |  | 2 | Yes | NA | 0 |
| COMCOM2 | *Neisseria macacae* | Co00804/3 |  | 2 | Yes | NA | 0 |
| COMCOM2 | *Neisseria macacae* | Co00804/3 |  | 2 | Yes | NA | 0 |
| COMCOM2 | *Neisseria mucosa* | Co00793/3 |  | 0.5 | Yes | NA | 0 |
| COMCOM2 | *Neisseria mucosa* | Co00795/2 |  | 2 | Yes | NA | 0 |
| COMCOM2 | *Neisseria mucosa* | Co00795/3 |  | 2 | Yes | NA | 0 |
| COMCOM2 | *Neisseria mucosa* | Co00796/4 |  | 2 | Yes | NA | 0 |
| COMCOM2 | *Neisseria mucosa* | Co00797/2 |  | 2 | Yes | NA | 0 |
| COMCOM2 | *Neisseria mucosa* | Co00801/1 |  | 1 | Yes | NA | 0 |
| MIC Control | *Neisseria gonorrhoeae* | ATCC 49226 |  | 0.125 | No | NA | 0 |
| MIC Control | *Neisseria gonorrhoeae* | WHO F |  | 0.06 | No | NA | 0 |
| MIC Control | *Neisseria gonorrhoeae* | WHO P |  | 0.25 | No | NA | 0 |
| MIC Control | *Neisseria gonorrhoeae* | WHO X |  | 0.06 | No | NA | 0 |
| MIC Control | *Neisseria gonorrhoeae* | WHO Z |  | 0.125 | No | NA | 0 |
| Pilot COMCOM2 | *Neisseria lactamica* | 001/E-SWAB |  | 0.5 | No | NA | 0 |
| Pilot COMCOM2 | *Neisseria macacae* | 001/OR |  | 2 | No | NA | 0 |
| Pilot COMCOM2 | *Neisseria macacae* | 002/OR |  | 2 | No | NA | 0 |
| Pilot COMCOM2 | *Neisseria macacae* | 003/E-SWAB |  | 1 | No | NA | 0 |
| Pilot COMCOM2 | *Neisseria mucosa* | 003/E-SWAB |  | 2 | No | NA | 0 |
| Pilot COMCOM2 | *Neisseria mucosa* | 003/OR |  | 4 | No | NA | 0 |
| PREGO | *Neisseria mucosa* | 19041762/2 |  | 2 | Yes | 0 | 1 |
| PREGO | *Neisseria mucosa* | 19051904/1 |  | 1 | Yes | 0 | 1 |
| PREGO | *Neisseria mucosa* | 19052394/2 |  | 2 | Yes | 0 | 1 |
| PREGO | *Neisseria mucosa* | 19062968/1 |  | 2 | Yes | 0 | 1 |
| PREGO | *Neisseria mucosa* | 19081774/2 |  | 2 | Yes | 0 | 1 |
| PREGO | *Neisseria mucosa* | 19111422/4 |  | 0.5 | Yes | 0 | 1 |
| Reference collection | *Neisseria cinerea* | ITM 2247 | ATCC 14685 | 0.25 | No | NA | 0 |
| Reference collection | *Neisseria lactamica* | ITM 2251 | ATCC 23970 | 0.25 | No | NA | 0 |
| Reference collection | *Neisseria lactamica* | ITM 3382 |  | 0.5 | No | NA | 0 |
| Reference collection | *Neisseria mucosa* | DSM 4631 |  | 0.5 | No | NA | 0 |
| Reference collection | *Neisseria mucosa* | ITM 1621 |  | 0.5 | No | NA | 0 |
| Reference collection | *Neisseria mucosa* | ITM 2252 | NCTC 19696 | 2 | No | NA | 0 |
| Reference collection | *Neisseria mucosa* | ITM 3369 | ATCC 19697 | 0.5 | No | NA | 0 |
| Reference collection | *Neisseria mucosa* | ITM 3375 | ATCC 19695 | 0.5 | No | NA | 0 |
| Reference collection | *Neisseria mucosa* | ITM 3380 | ATCC 19694 | 1 | No | NA | 0 |
| Reference collection | *Neisseria mucosa* | ITM 3391 | ATCC 25999 | 0.5 | No | NA | 0 |
| Resistogenicity study | *Neisseria macacae* | MO00012/2 |  | 1 | Yes | 0 | SRR13753794 |
| Resistogenicity study | *Neisseria macacae* | MO00018/2 |  | 1 | Yes | 0 | SRR13753792 |
| Resistogenicity study | *Neisseria macacae* | MO00029/2 |  | 1 | Yes | 0 | SRR13753793 |
| Resistogenicity study | *Neisseria subflava* | ITM45/1 |  | 2 | Yes | 0 | SRR13753781 |
| Resistogenicity study | *Neisseria subflava* | ITM9/1 |  | 0.5 | Yes | 0 | SRR13753790 |

STable 2. Identity and zoliflodacin MICs of *N. gonorrhoeae* strains used for intraspecies transformation experiments

| Recipient strain | | |  | Donor strain | |  | Transformant | |
| --- | --- | --- | --- | --- | --- | --- | --- | --- |
| Species | Sample_ID | Initial MIC (µg/mL) | Sample_ID | Final MIC (µg/mL) | GyrB substitution | nucleotide (nr of reads (%)) | *gyrB* mutation transformant (nr of reads (%)) | MIC (µg/mL) |
| *Neissera gonorrhoeae* | ATCC49226 | 0.25 | ATCC49226_8 | 8 | S467N | G1400A (1107/1109 (100%) | No growth | NA |
| *Neisseria gonorrhoeae* | WHO P | 0.125 | WHO P_8 | 8 | M29I | G87A (1481/1484 100%) | No growth | NA |

NA - not applicable

**Supplementary Figure 1**

**
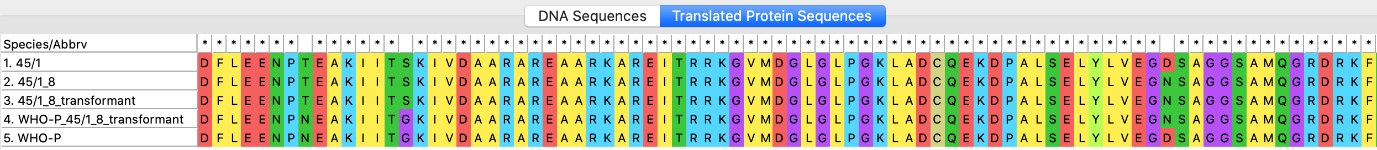
**

SFigure1. Sequence alignment of partial *gyrB* gene of the *N. subflava* baseline strain 45/1 (reference), after zoliflodacin resistance induction (45/1-8), after transformation with its own resistant strain (45/1_8_transformant) and transformation of the amplicon in *N. gonorrhoeae* strain WHO P (WHO-P_45/1_8_transformant). The sequence alignment is based on the deduced amino acids using clustalW implemented in MEGA-X (V 2.0). The black arrow indicates amino acid position 429.
